# Immunogenicity and Loss of Effectiveness of Biologic Therapy for Inflammatory Bowel Disease Patients Due to Anti-Drug Antibody Development

**DOI:** 10.3390/antib13010016

**Published:** 2024-02-26

**Authors:** Tsvetelina Velikova, Metodija Sekulovski, Monika Peshevska-Sekulovska

**Affiliations:** 1 Medical Faculty, Sofia University St. Kliment Ohridski, 1 Kozyak Str., 1407 Sofia, Bulgaria; tsvelikova@medfac.mu-sofia.bg (T.V.); metodija.sekulovski@gmail.com (M.S.); 2 Department of Anesthesiology and Intensive Care, University Hospital Lozenetz, 1 Kozyak Str., 1407 Sofia, Bulgaria; 3 Department of Gastroenterology, University Hospital Lozenetz, 1407 Sofia, Bulgaria

**Keywords:** inflammatory bowel disease, biologic therapy, antibodies to biologics, anti-TNF antibodies, TNF antagonist, anti-TNFa, infliximab, treatment response failure, immunogenicity, loss of effectiveness, safety

## Abstract

Many patients with inflammatory bowel disease (IBD) experience a loss of effectiveness to biologic therapy (i.e., anti-TNF therapy, etc.). Therefore, in addition to the adverse effects of the treatment, these patients also face failure to achieve and maintain remission. Immunogenicity, the process of production of antibodies to biological agents, is fundamental to the evolution of loss of response to treatment in IBD patients. The presence of these antibodies in patients is linked to decreased serum drug levels and inhibited biological activity. However, immunogenicity rates exhibit significant variability across inflammatory disease states, immunoassay formats, and time periods. In this review, we aimed to elucidate the immunogenicity and immune mechanisms of antibody formation to biologics, the loss of therapy response, clinical results of biological treatment for IBD from systematic reviews and meta-analyses, as well as to summarize the most recent strategies for overcoming immunogenicity and approaches for managing treatment failure in IBD.

## 1. Introduction—Challenges Related to the Use of Biologic Therapy in IBD Patients

Biologic therapy for treating inflammatory bowel disease (IBD) patients started optimistically 25 years ago [1]. Since then, approvals for clinical use for IBD have been authorized for five anti-TNF drugs (infliximab, adalimumab, golimumab, certolizumab pegol), along with biosimilars [2], and for anti-IL-12/IL-23 (ustekinumab), anti-α4β7-integrin (vedolizumab), and for some countries, for anti-α4-integrins (natalizumab), etc., while other treatments have been implemented for other autoimmune disorders and discussed as having potential efficacy for IBD—etanercept, anti-IL-17 (secukinumab, ixekizumab, brodalumab), etc. Additionally, more recently developed small molecules for the treatment of IBD and other inflammatory diseases have been introduced (i.e., JAK inhibitors (tofacitinib)) [3], although they are not labeled as “biologics”.

However, along with their undeniable effectiveness in enabling IBD patients to achieve and maintain disease remission, biological therapies face some challenges regarding safety, loss of effectiveness, high price, etc. The first and most significant challenge when using biologics as monotherapy in IBD management is that only a maximum of 40% of patients achieve a state of remission at the end of the first year of therapy [4].

Furthermore, patients with poor response to therapy or refractory IBD, including having extraintestinal complications, are often offered dual biologic therapy [3].

Recent systematic reviews and meta-analyses showed that the most common adverse effects of biologics used as monotherapy in IBD are arthralgia, flares, and skin lesions (i.e., eczema, psoriasis), and the most severe—infections (odds ratio, OR 0.89–3.60 in different meta-analyses, OR 1.90 for opportunistic infections) and malignancies (with OR for older people (OR = 3.07)—higher than for younger biologic users) [5,6,7].

Additional issues related to the optimal sequence biological therapy for IBD are still challenging. Many studies for ulcerative colitis (UC), such as GEMINI 1, VARSITY, ULTRA 2, and True North, showed lower clinical remission rates with adalimumab, vedolizumab, and ozanimod after anti-TNF therapy, while other studies, such as OCTAVE 1, 2, U-ACHIEVE/U-ACCOMPLISH, and UNIFI (ustekinumab, tofacitinib, and upadacitinib) did not. At the same time, the EXTEND and GEMINI 1, 2 projects in Crohn’s disease (CD) patients showed that adalimumab and vedolizumab are associated with persistent lower endoscopic remission after anti-TNF therapy, but ustekinumab and risankizumab (from IM-UNIFI, FORTIFY and SEAVUE) do not result in remission. On the other hand, EVOLVE demonstrated that vedolizumab could be used as a first-line biologic because it does not impact further anti-TNF therapy [8]. Therefore, the controversial outcomes regarding the optimal sequence of biologics for IBD emphasize the need to explore this issue more.

Another issue related to IBD treatment is the development of new agents. Zurba et al. explored the pipeline of novel therapies for IBD. The authors also discussed the major issues associated with biologics for IBD, namely, primary non-response, secondary loss of response, and adverse effects (short- and long-term) [9]. However, there are novel treatment approaches, such as modulation of host-microbiome interactions, stem cell therapy, fibrosis management, gut-brain axis modulation, and targeted B cell therapy. Still, a definitive therapy or long-term remission for IBD is likely not realistic at this stage of the science [10].

Nonetheless, advances in the medical care of IBD have risen in recent years, boosted by the innovative small molecule and novel biologic medicines discussed here. Although the observed clinical response remains sub-optimal, treatment options for IBD patients are fast evolving to help address the disease burden, morbidity, mortality, and quality of life. In the absence of large-scale trials, physician experience has now led to prospective innovative therapeutic combinations, with most data described in case reports and case series [11].

Despite these advance, the place and future of biologics remain challenging to determine. They and other advanced therapies (i.e., novel small molecules) have made considerable contributions in providing personalized treatment for IBD patients [12]. The problems related to choosing the optimal single-agent, sequential therapy following treatment failure, dual biologic therapy, and small molecules were recently addressed by the British Society of Gastroenterology, which issued consensus guidelines on IBD management in adults [13].

Another unresolved problem associated with biologics is how to discontinue therapy. Miyatani & Kobayashi recommended discontinuation based on the evidence for the risk of relapse and efficacy of re-treatment, where the risk of relapse is higher when ceasing the anti-TNF drugs. However, in the case of withdrawal of immunomodulators combined with biologics (i.e., anti-TNFa), there is a need for therapeutic drug monitoring [14].

Notwithstanding, the determination of the best approach for a new or bio-naïve IBD patient is also an issue. In this case, along with the information from the clinical trials, factors such as comorbidities, genetic background, inflammatory markers, patient preferences, cost of the therapy, etc., should be taken into account [15]. Another debating issue concerns the implementation of biologics early in IBD treatment management. Most evidence supports the early administration of biologics in CD to improve outcomes and prevent complications and disease progression, but not for UC [16]. A meta-analysis by Ben-Horin et al. supported the same observation and recommended early administration of biologics for CD, but not for UC [17].

In this review, we aimed to elucidate the immunogenicity and immune mechanisms of antibody formation to biologics, the loss of therapy response, clinical results of biological treatment for IBD from systematic reviews and meta-analyses, as well as to summarize the most recent strategies for overcoming immunogenicity and approaches for managing treatment failure in IBD.

## 2. Search Strategy

We conducted a modified narrative review on the topic by first searching through scientific literature (bibliographic databases Medline (PubMed), Scopus, Cochrane Central Register of Trials (CENTRAL), and Cochrane Database of Systematic Review (CDSR). Relevant free-text and MeSH terms were used: (“biologics” OR “biologic therapy”) AND (“inflammatory bowel disease” OR “IBD” OR “ulcerative colitis” OR “Crohn’s disease”) AND (“loss of efficacy”) AND (“immunogenicity”). Additionally, we searched for (“anti-TNFa” OR “anti-TNFa drug”) AND (“loss of efficacy”), (“biologics” OR “biologic therapy”) AND (“withdrawal” OR “discontinuation”). We also searched for (“immunogenicity” OR “anti-drug antibodies”) AND (“adalimumab” OR “ADM”)/(“certolizumab pegol” OR “CZP”)/(“golimumab” OR “GLM”)/(“infliximab” OR “IFX”)/(“ustekinumab” OR “UST”)/(“vedolizumab” OR “VDM”).

In addition to the mentioned databases, we searched these terms through Google Scholar and also for supplements. We searched for appropriate papers and wrote the review according to the recommendations [18].

**Figure 1:**
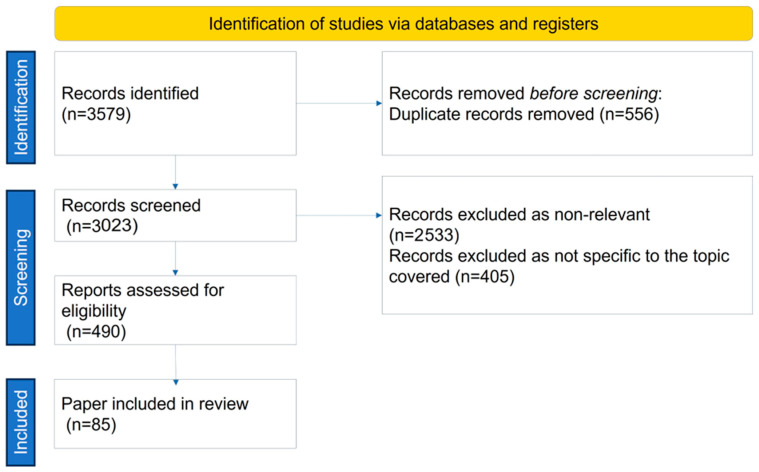
Search strategy.

The search strategy process, including the number of papers found, excluded, and reasons for exclusion, and included, is shown in Figure 1.

## 3. Biologic Therapy—Tailoring the IBD Pathogenetic Mechanisms

The current research focuses on elucidating the immunological mechanisms and the loss of response to various categories of biological agents for treating IBD. However, to better understand these mechanisms, it is essential to underline that biologics are monoclonal antibodies (MAbs), that is, therapeutic immunoglobulins G (IgG) with four polypeptide chains with two heavy and two light chains and two functional regions: the variable (antigen-binding region, Fab) and the constant region (Fc). The nomenclature of the most common mAbs used in IBD therapy are based on their derivation: murine (-omab), chimeric (-ximab), humanized (-zumab), and entirely human (-umab). An understanding of the pathophysiological mechanisms of IBD is critical before discussing immunogenicity [19].

Although the pathogenetic processes in IBD are not entirely comprehended, they undoubtedly influence both the therapeutic effectiveness and adverse effects of biological agents.

**Figure 2:**
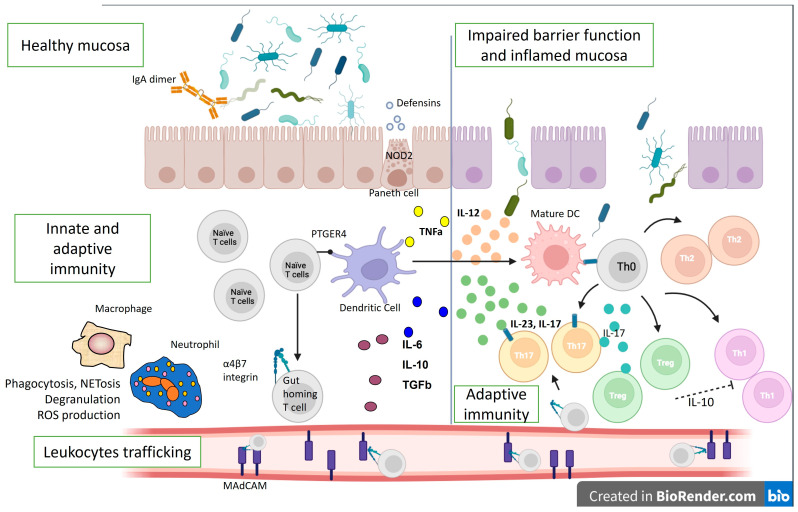
Immunological mechanisms in IBD pathogenesis. The lines represent differentiation, and the dotted line—suppression. IL—interleukin; ROS—reactive oxygen species; Th—T helper cell; DC—dendritic cell; TGFb—transforming growth factor beta; PTGER4—Prostaglandin EP4 receptor; NOD2—Nucleotide Binding Oligomerization Domain Containing 2; TNFa—tumor necrosis factor-alpha; MAdCAM—Mucosal vascular addressin cell adhesion molecule 1.

In healthy mucosa, the mucus layer and epithelial cells maintain barrier function. The intestinal epithelium, along with Paneth cells, which produce antimicrobial peptides, and M cells, which sample lumenal antigens and IgA dimers, help regulate and separate lumenal bacteria from the mucosal immune system. Dendritic cells (DCs) also sample lumenal contents to maintain immunologic tolerance in the intestine through podocytes across the epithelium. They process and deliver antigens to T and B lymphocytes that reside in the draining lymph nodes to induce immune tolerance [20]. Intestinal dendritic cells can stimulate naïve T and B lymphocytes to express the gut-homing marker α4β7 (Figure 2).

Intestinal lymphocytes imprinted with α4β7 interact with locally generated MAdCAM to re-enter the intestinal lamina propria and avoid circulation. Additionally, the intestinal lamina propria contains Th1, Th17, and Treg cells. The gut mucosa maintains balance through coordinated innate and adaptive immune cells, where Treg cells regulate Th1 and Th17 cells, reducing inflammation [21].

Both innate and adaptive immune mechanisms are also involved in IBD pathogenesis. Allelic variations in NOD2 have faulty intracellular bacteria sensing and reduced defensin synthesis by Paneth cells in the base of the intestinal crypts in the mucosa of CD patients. The result is an increased adaptive immune response to compensate for ineffective innate immunity. The interleukin (IL)-12/IL-23 pathway can also disrupt adaptive immunity, shifting the helper T-cell response to the Th17 spectrum. Ustekinumab, which blocks the p40 component of IL-23 and IL-12, was shown to be effective in CD.

Additionally, Th1 and Th17-associated inflammation outweighs Treg regulation by making Tregs ineffective and suppressed [22], complicating the pathophysiological picture. However, many other immune cells and cytokines are involved in the pathogenesis of IBD [23]. For example, in IBD patients, DCs and macrophages are activated during inflammation and secrete large amounts of mucosal TNF and other mediators. This pleiotropic cytokine has several pro-inflammatory effects, and anti-TNF antibodies (i.e., infliximab, adalimumab, certolizumab, golimumab) are among the biologics that are used in the treatment of CD and UC. Furthermore, when exposed to MHC class II antigens and a co-stimulatory signal, macrophages and DCs activate T lymphocytes, involving adaptive immune mechanisms.

Additionally, the receptor variant PTGER4 can cause intestinal mucosa barrier defects that promote microbial and antigenic penetration and immune activation [24]. Mucosal immune response amplification requires leukocyte movement. α4-integrins (α4β1 and α4β7) bind to ICAM-1 in inflamed tissues and MAdCAM-1, which is particular to the intestinal endothelium. In line with this, homing inhibitors, such as vedolizumab, natalizumab, and etrolizumab, disrupt inflammation by blocking inflammatory cell adhesion and recruitment [25]. Moreover, a breach in the epithelial mucosal barrier permits lumenal bacteria to cause uncontrollable inflammatory responses in UC. Th9 inflammatory cells further promote enterocyte death and hinder mucosal repair, and NKT cells generate IL-13, which damages epithelial cells. Cytokines secreted by the innate lymphoid cells (ILCs) also contribute to inflammation. Therefore, ILCs are significant mediators of chronic intestinal inflammation and drivers of disease pathogenesis, making them targets for prospective novel therapeutics, such as the JAK pathway inhibitor, tofacitinib [26].

**Figure 3:**
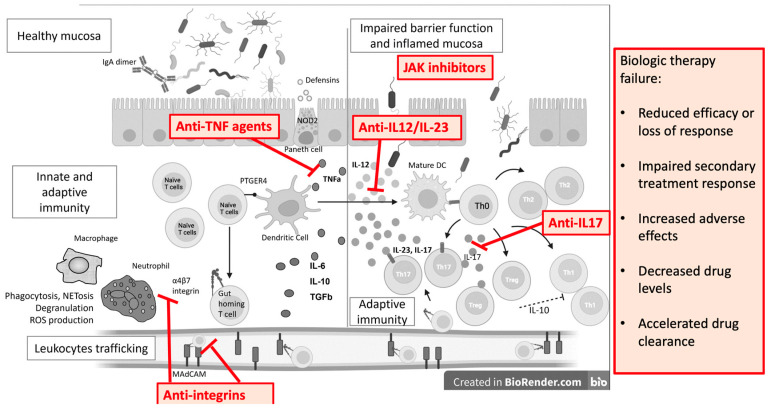
Monoclonal antibodies and small molecules in IBD treatment and causes of treatment failure. IL—interleukin; ROS—reactive oxygen species; Th—T helper cell; DC—dendritic cell; TGFb—transforming growth factor beta; PTGER4—Prostaglandin EP4 receptor; NOD2—Nucleotide Binding Oligomerization Domain Containing 2; TNFa—tumor necrosis factor alpha; MAdCAM—Mucosal vascular addressin cell adhesion molecule 1.

On the other hand, dysbiosis also causes mucosal damage and inflammation. Due to a better understanding of the gut immune system, the development of novel therapeutic targets has expanded. TNFα antagonists, integrin inhibitors, anti-IL-12/23 inhibitors, and JAK inhibitors are now in clinical use, and others are in early to advanced phases of development (Figure 3). However, we must always consider that immunological molecules and cells work together in a network where regulation is essential.

## 4. Immunological Mechanisms of Biological Therapy Failure

The most common cause of failure of biological therapy is the development of anti-drug antibodies. Other immunological mechanisms of the loss of efficacy of biological treatment are empirically confirmed by data showing that the concomitant use of immunosuppressive drugs reduces the immunogenicity and overall antibodies to drug production [27,28]. Indeed, studies showed that at least 30% of patients fail to meet primary endpoints and other patients experience a loss of efficacy over time [29,30,31].

Since biologics are large and complex protein molecules, they are highly immunogenic and usually initiate immune responses towards them. Immune responses include activating T and B cells and eventually producing antibodies specific to the drug [32].

In the case of the development of antibodies against a drug, the clinical consequences are usually associated with loss of efficacy. Therefore, IBD patients with such a loss of response to therapy should be switched to a second anti-TNFa agent (i.e., adalimumab after the loss of response to infliximab or the opposite), or a different agent (preferably with another mechanism of action) [33,34].

However, we must acknowledge that based on the distinct methodologies and assay techniques (i.e., ELISA, RIA, ECLIA, etc.) performance, the presence of anti-drug antibodies is estimated at different levels in the studies [32,35,36,37]. The review of Vermeire et al., (2018) showed that the formation rates of anti-drug antibodies varied significantly among the studies: for infliximab—0–65.3% (73 studies), adalimumab—0.3–38% (22 studies), certolizumab pegol—3.3–25.3% (four studies), vedolizumab—1–4.1% (four studies), golimumab—0.4–2.9 (two studies), ustekinumab—0.7% (one study) [38]. Similarly, a recent systematic review and meta-analysis by Bots et al., (2023), which included 68 studies and 5850 patients, revealed pooled rates of antibodies to biologics as follows: infliximab—28%, adalimumab—7.5%, golimumab—3.8%, certolizumab—10.9%, ustekinumab—6.2%, natalizumab—16%, verdolizumab—8.4% and etrolizumab—5% [39]. We can see that the rates are similar in the two meta-analyses, although they differ depending on the current data at the moment of analysis and the included studies. Nevertheless, the antibody formation rates are as high as one-third to more than half of the patients.

Vermiere et al. also summarized the most influential factors for developing antibodies against biologics, divided into three main groups: (a) related to the drug (structure of the molecule, duration of treatment, route of administration, combinations with other drugs (i.e., immunosuppressants); (b) related to the individual (age, sex, genetic background, underlying diseases, prior exposure to biologics, immune competence, etc.); (c) related to the measurement techniques (type of assay, timing of sampling and drug exposure) [38].

Regarding genetics, it is still unclear which genes and mechanisms are related, but some studies also confirmed the role of genetic factors in developing immunogenicity [40].

The authors also discussed the possible role of anti-drug antibody formation on the treatment efficacy, safety, pharmacokinetics, and the overall course of immunogenicity [38,41,42,43,44,45,46,47,48,49,50,51,52] (Table 1).

Speaking of the delicate mechanisms of action of antibodies against biologics, it was shown that antibodies to TNF inhibitors may exert two effects depending on the binding site. The agent therapeutic activity is reduced when the anti-drug antibodies bind to the epitope (Fab’) 2 region of the anti-TNF monoclonal antibody, thus preventing the binding of the drug to the target molecule (i.e., TNFa). In this case, the anti-drug antibodies are neutralized [53].

In contrast, non-neutralizing antibodies do not directly reduce the efficacy of the biological drug because they do not affect the epitope binding. Hence, these non-neutralizing antibodies can hamper the pharmacokinetics by facilitating drug clearance [54]. We also must remember that some anti-drug antibodies are transient and do not have clinical significance, unlike persistent antibodies. Thus, they can rarely lead to efficacy loss and treatment failure [55,56,57,58,59].

## 5. Recent Systematic Reviews and Meta-Analyses on Biologic Failure in IBD Patients

Gisbert et al., [60] analyzed 46 papers, consisting of 37 studies focused on CD, eight on UC, and one on pouchitis. The clinical trials included a total of 32 patients who switched from infliximab (IFX) to adalimumab (ADA), four patients who switched from IFX to certolizumab pegol (CZP), and one patient who switched from ADA to IFX. In general, administering a second anti-tumor necrosis factor (TNF) agent following infliximab (IFX) ineffectiveness in patients with CD resulted in remission in 43% of individuals. It elicited a response from 63% of the patient population. The rate of remission was found to be higher in cases where the first anti-TNF treatment was discontinued due to intolerance (61%) compared to cases where it was discontinued due to secondary (45%) or primary failure (30%). The corresponding response rates were 72%, 62%, and 53%, respectively. Among trials conducted at the University of California, six reported varying remission percentages, ranging from 0% to 50%. The incidence of adverse events in individuals with CD varied between 0 and 81%, with most of these occurrences classified as mild. Serious adverse events were reported from 0 to 21%, and the discontinuation rate due to adverse events was less than 20% [60].

The effectiveness of a second anti-tumor necrosis factor (TNF) treatment in patients with CD is predominantly contingent upon the underlying reason for transitioning to an alternative therapy. The rate of remission is shown to be higher in cases where the first anti-TNF treatment is discontinued due to intolerance (61%), as opposed to cases where it is terminated due to secondary (45%) or primary failure (30%). Additional research is required to assess the efficacy of transitioning from adalimumab (ADA) to infliximab (IFX) as a therapeutic approach [60], as well as other switches in treatment.

As for the other biologics, such as anti-integrins, there are fewer data on loss of effectiveness or primary non-response. The systematic review by Attauabi et al., (2022) included 2830 (bio-naïve UC patients) and 2381 (bio-naïve CD patients), compared with 7392 (UC) and 10,511 (CD) bio-exposed patients [61]. They established that bio-naïve UC patients had higher rates of clinical remission after vedolizumab at week 14 (RR = 1.27 [95% CI 1.00, 1.62]) and week 52 (RR = 1.25 [95% CI 1.11, 1.42]) compared to bio-exposed patients. Steroid-free clinical remission at week 52 was similar (RR = 1.36 [95% CI 1.06, 1.76]). Furthermore, this study demonstrated that bio-naïve CD patients had a higher chance of clinical remission at week 52 (RR = 1.23 [95% CI 1.05, 1.43]) but not at week 14 or steroid-free [61].

Nevertheless, studies showed a more favorable safety profile of vedolizumab than other biologics. Six clinical trials involving more than 2800 subjects documented no increased risk of infections, including from opportunistic agents or severe infections (i.e., listeria meningitis, clostridial infections, tuberculosis, sepsis, etc.), nor any increased risk of malignancies [62,63].

However, the effectiveness of vedolizumab (remission and clinical response) is inversely correlated with the initial levels of systemic and intestinal inflammation [64,65].

Peyrin-Biroulet et al., in their systematic review, demonstrated the pooled incidence rates of loss of response as 47.9/100 person-years of follow-up for CD patients and 39.8/100 person-years of follow-up in UC patients. Additionally, dose intensification was able to restore response to the drug in 53.8% of patients with lost effectiveness (secondary non-responders) [66].

The FDA-approved for psoriasis IL-17 inhibitors, Cosentyx (secukinumab), Taltz (ixekizumab), and Siliq (brodalumab), can also lose their efficacy due to loss of response [67,68]. However, recently, an IL-17 inhibitor-associated IND was described [69].

Other anti-cytokine therapies approved for IBD are anti-IL-12/23 monoclonal antibodies [70].

There are some differences related to anti-IL-23 ustekinumab biologics. First, the rate of anti-drug antibodies is low (4.6% through 156 weeks of therapy), regardless of the use of additional immunomodulators (i.e., azathioprine, 6-mercaptopurine, or methotrexate) or without (5% vs. 4.5%) [71,72].

Most importantly, the developed anti-drug antibodies were not associated with loss of effectiveness, since they are not neutralizing antibodies.

The third generation of anti-IL-23 represents synthetic small molecules with many advantages over other biologics. An oral delivery form is preferable by most patients and is less immunogenic, i.e., no concern of secondary nonresponse. Nevertheless, small molecule pharmacokinetics allow all of the drug to be absorbed, even in severely inflamed patients with protein leakage [73].

Other significant research on IBD biologics loss of effectiveness was performed by State & Negreanu (2023), who conducted a systematic review to define the failure of advanced therapies in IBD [74]. The authors pointed out a lack of evidence on treatment optimization, treatment failure, and criteria to abandon or switch to other treatments, primarily based on the heterogeneity of the studies and the outcomes reported. This lack of clear definitions and official recommendations carries risks for empirical therapy and early abandonment of biologics [74]. Taken together, data showed that biologics could be effective in achieving clinical remission, but long-term efficacy is hardly achievable.

## 6. Strategies for Precise Detection and Preventing Immunological Failure of IBD Biologics Due to Antibody Formation

One of the best approaches to lower the production and impact of antibodies to biological drugs is using immunosuppressants simultaneously with biologics [75,76]. A meta-analysis by Garces et al. demonstrated that methotrexate use along with infliximab or adalimumab reduced the proportion of antibodies to these drugs by 41% in patients with IBD and rheumatic diseases [28]. Similarly, Jani et al. discussed the potential of immunosuppressive drugs to reduce the production of antibodies to biologics, although the mechanisms behind this remain unclear [27].

In addition to these observations are the approaches for scheduling treatment with infliximab that lead to improved efficacy and reduced immunogenicity compared to episodic treatment [77,78]. Bots et al., (2021) demonstrated reduced rates of anti-drug antibodies in IBD patients treated with combination therapy consisting of infliximab (RR 0.52), adalimumab (RR 0.31), golimumab (RR 0.29), certolizumab pegol (RR 0.30), and natalizumab (RR 0.20) [39].

Administration of a loading dose of a biologic agent at the initiation of therapy as well as corticosteroids i.v. before infusion with infliximab also showed reduced immunogenicity [45,79]. Adding immunomodulators to the biological treatment could also benefit the patients by lowering the levels of already-produced anti-drug antibodies. However, the optimal doses of these immunomodulators that could inhibit the production of antibodies to biological agents are not established, although Roblin et al. demonstrated that lower doses of azathioprine are enough to prevent antibody production to infliximab [41,50,80,81,82,83,84,85].

The drug administration route could also influence the production of antibodies to the drug. For example, Schreiber et al. reported that subcutaneous administration of infliximab is less immunogenic than infusions [86]. Some investigators established that higher dosing of TNF inhibitors could also lead to a decline in antibodies to biological agent antibodies [87,88]. However, we must remember that higher doses of TNF inhibitors could also mask the detection of antibodies against the drug.

Kothari et al., (2017) proposed an algorithm for managing the secondary loss of response, recommending that when the drug level is low, along with no/low levels of anti-drug antibodies, the drug dose should be increased. However, if the antibodies are at a higher titer, the therapy should be changed (within the class or alternate class). The authors demonstrated that by using these strategies, most patients could resolve the antibodies to TNF antagonists while improving disease activity scores [89]. Furthermore, other studies also showed that low levels of antibodies could be overcome [56,80,90].

Moss gave an opinion on the approaches to treatment failure in IBD in 2022 [91], focusing on factors such as multiple options available for treating IBD patients, measuring drug antibodies to predict losing response to therapy, escalating the dose or switching to another agent, deciding on whether the treatment failed when the induction is over, inflammatory and non-inflammatory factors for failure to respond to therapy, risk factors for failure of treatment (i.e., deep ulcers, high CRP, low serum albumin levels, high inflammatory state of disease, etc.), optimizing biological therapy, remission rates after therapeutic failure, the need for surgery after therapy failure, etc. [91].

However, some gaps in the knowledge are still present. How the titers of anti-drug antibodies correlate with drug concentrations is unclear. Still, it is a challenge when the therapy is low-dose, and the antibodies to the drug could present at very low levels, thus undetectable on the assays. Probably, drug-tolerant assays could be helpful in these cases, although the immunogenicity rates for some of the anti-TNF drugs are higher (i.e., infliximab, vedolizumab, golimumab) [87,92,93,94].

Switching to biosimilars could benefit some patients, based on the data that this approach is safe, effective, and not linked to increased immunogenicity [95,96].

A risk-based strategy is needed to overcome the immunogenicity associated with using TNF antagonists and other biopharmaceuticals. On the one hand, in the case of antibody production, the levels should be evaluated precisely and then clinically correlated case-by-case before deciding on patient treatment. On the other hand, long before launching a drug in clinical trials, in silico and in vitro techniques must be employed to identify and eliminate putative T cell epitopes (studied in the HLA context) while maintaining the structure and function of the molecule candidate. However, predicting the immunogenicity of potential therapeutic proteins is still challenging [97].

## 7. Conclusions

In conclusion, the challenges encountered by individuals with IBD receiving biologic therapy, particularly anti-TNF therapy, are multiple. The loss of treatment effectiveness presents a significant obstacle, further compounded by the potential adverse effects of the therapeutic regimen. The intricate interplay between immunogenicity and the development of antibodies against biological agents stands as a pivotal factor in understanding the emergence of treatment failure among IBD patients. These antibodies directly impact the serum drug concentrations, leading to attenuation of biological activity. However, the immunogenicity rates exhibit noteworthy heterogeneity across diverse inflammatory disease contexts, distinct immunoassay formats, and varying timeframes.

Moreover, there are still gaps in the knowledge of resistance to biologics for IBD patients; the three main deficiencies being differential immunogenicity profiles, the long-term impact of immunogenicity, and the lack of clear guidelines on the optimal switching strategies after biological failure. In line with this, addressing the identified gaps in knowledge is paramount to unraveling the intricacies of anti-drug resistance in IBD, providing a foundation for targeted research endeavors and paving the way for innovative strategies to enhance patient therapeutic outcomes. Investigating why certain individuals develop anti-drug antibodies against specific biologics while others do not is essential for personalized treatment strategies. Additionally, exploring whether sustained anti-drug antibodies correlate with disease progression, loss of response, or adverse events over extended periods is pivotal for optimizing treatment regimens. Identifying the most effective subsequent biologic or non-biologic therapeutic options, while considering individual patient profiles and drug mechanisms, is crucial for minimizing treatment gaps and maximizing positive outcomes in IBD management. Furthermore, there are advancements in strategies to mitigate immunogenicity, propounding innovative approaches to contend with treatment setbacks encountered by patients with IBD.

## Figures and Tables

**Table 1:** Different aspects of biological therapy for IBD due to antibodies to drug formation or other mechanisms.

Aspects	Consequences	References
Treatment efficacy	Reduced efficacy, assessed by CDAI, Mayo score, endoscopic improvement, immunological parameters, etc. Failure to achieve remission Loss of response High rate of secondary treatment failure (83.6%) Lower clinical response rate for IFX	Vermeire et al. [38]; Karmiris et al. [41]
Treatment safety	More common adverse events for IFX Higher risk of adverse events and infusion reactions in re-retreatment with the same agent after a break in treatment (cease of maintaining therapy) Higher rate of infusion-related reactions for IFX No increased safety issues for ADM, CZP, GLM, VDM, and UST. Worsening of the patient’s condition (i.e., increased CRP, fecal calprotectin, etc.)	Vermeire et al. [38]; Bots et al. [39]; Baert et al. [42]; Casanova et al. [43]; Torres et al. [44]; Farrell et al. [45]; Vande Casteele et al. [46]; Sandborn et al. [47];
Treatment pharmacokinetics	Decreased drug levels in patients with anti-drug antibodies (observed for ADM, CZP, and IFX) Low serum drug concentration due to accelerated clearance Switching from one to second anti-TNF drug is associated with a higher risk of anti-drug formation.	Vermeire et al. [38]; Brandse et al. [48]; Papamichael et al. [49]; Roblin et al. [50]; Bartelds et al. [51]
Timing of immunogenicity	Antibodies production as early as 10–14 days post-infusion Weeks or months after the first, second, third, etc. infusions to reach detectable levels Sampling timing affects the detection rate: RCTs report lower rates of anti-drug formation vs. observational studies.	Vermeire et al. [38];
Pharmacoeconomics	Monitoring antibodies to IFX in CD patients is cost-effective instead of dose-escalating.	Vermeire et al. [38]; Steenholdt et al. [52]

CDAI—Crohn’s Disease Activity Index, IFX—infliximab, ADM—adalimumab, CZP—certolizumab pegol, GLM—golimumab, UST—ustekinumab, VDM—vedolizumab, CD—Crohn’s disease, RCT—randomized clinical trial.
